# AMPK activation eliminates senescent cells in diabetic wound by inducing NCOA4 mediated ferritinophagy

**DOI:** 10.1186/s10020-024-00825-8

**Published:** 2024-05-17

**Authors:** Mengqian Liu, Xuerong Wei, Zijun Zheng, Erlian Xie, Qiuyi Yu, Yanbin Gao, Jun Ma, Lei Yang

**Affiliations:** Department of Burns, Nanfang Hospital, Southern Medical University, Jingxi Street, Guangzhou, 510515 Guangdong China

**Keywords:** Diabetic wound, Cellular senescence, Ferroptosis, Autophagy, AMPK

## Abstract

**Background:**

Diabetic wounds are one of the long-term complications of diabetes, with a disordered microenvironment, diabetic wounds can easily develop into chronic non-healing wounds, which can impose a significant burden on healthcare. In diabetic condition, senescent cells accumulate in the wound area and suppress the wound healing process. AMPK, as a molecule related to metabolism, has a close relationship with aging and diabetes. The purpose of this study was to investigate the effects of AMPK activation on wound healing and explore the underlying mechanisms.

**Methods:**

AMPK activator A769662 was topically applied in wound models of diabetic mice. Alterations in the wound site were observed and analyzed by immunohistochemistry. The markers related to autophagy and ferritinophagy were analyzed by western blotting and immunofluorescence staining. The role of AMPK activation and ferritinophagy were also analyzed by western blotting.

**Results:**

Our results show that AMPK activation improved diabetic wound healing and reduced the accumulation of senescent cells. Intriguingly, we found that AMPK activation-induced ferroptosis is autophagy-dependent. We detected that the level of ferritin had deceased and NCOA4 was markedly increased after AMPK activation treatment. We further investigated that NCOA4-mediated ferritinophagy was involved in ferroptosis triggered by AMPK activation. Most importantly, AMPK activation can reverse the ferroptosis-insensitive of senescent fibroblast cells in diabetic mice wound area and promote wound healing.

**Conclusions:**

These results suggest that activating AMPK can promote diabetic wound healing by reversing the ferroptosis-insensitive of senescent fibroblast cells. AMPK may serve as a regulatory factor in senescent cells in the diabetic wound area, therefore AMPK activation can become a promising therapeutic method for diabetic non-healing wounds.

**Supplementary Information:**

The online version contains supplementary material available at 10.1186/s10020-024-00825-8.

## Introduction

Chronic non-healing wounds are one of the long-term complications of diabetes, which place a huge burden on global public health (Falanga 2005). Diabetic wounds exhibit a complex microenvironment and the exact molecular mechanism of diabetic non-healing wounds is still far from fully understood (Baltzis et al. 2014). Previous studies have reported that the percentage of senescent cells is significantly increased at the wound site of diabetic patients, and the overaccumulation of senescent cells is correlated with an impaired wound healing process (Tomic-Canic and Di Pietro 2019).

AMPK, a heteromer complex consisting of three subunits, is an important kinase for regulating energy metabolism and a key protein involved in multiple signal transduction pathways (Salminen and Kaarniranta 2012). Recent studies show that AMPK plays a vital role in regulating senescent cell function, and AMPK activation has a positive effect on aging-related diseases, especially in diabetic patients (Jeon 2016; Entezari et al. 2022). In diabetic patients, the chronic inflammatory condition suppresses AMPK activation and restricts several AMPK-regulated pathways (Salminen et al. 2008). Multiple studies have verified that AMPK activation can influence the pathological process of diabetes. In diabetic kidney disease, AMPK activation can alleviate diabetic renal tubular injury by alleviating mitochondrial fission via AMPK/SP1/PGAM5 pathway (Liu et al. 2020). In a study on diabetic endothelial damage, AMPK activation can protect against high glucose induced injury by inhibiting endothelial-mesenchymal transformation (Mao et al. 2022). Although intensive studies have illustrated that systematically regulating AMPK has a positive effect on diabetes, there are few reports focusing on the topical treatment of AMPK activation in diabetic wounds.

AMPK plays a vital role in programmed cell death (Kandula et al. 2022). Upregulation of AMPK can regulate cellular redox homeostasis and induce ferroptosis in a gestational diabetes mellitus model (Han et al. 2020). Ferroptosis is a novel type of programmed cell death that is iron-dependent and causes lipid peroxidation via Fenton reaction (Dixon et al. 2012). In recent years, several studies have considered ferroptosis as an autophagy-dependent mode of death (Ajoolabady et al. 2021). The process of ferroptosis is tightly related to the intracellular iron level, and ferritin is the primary molecule that regulates iron metabolism (Bogdan et al. 2016). Autophagy activation can degrade ferritin and release free iron, which leads to oxidative damage resulting from the Fenton reaction (Hou et al. 2016). Nuclear receptor coactivator 4 (NCOA4) is a selective receptor for ferritin, which can transport ferritin into lysosomes for autophagic degradation (Mancias et al. 2014). This process is termed ferritinophagy and regulates the intracellular iron homeostasis.

AMPK is the main kinase involved in the process of autophagy, which enhances the expression of autophagy transcription factors such as FOXOs by regulating mTORC1 and ULK1, consequently promoting autophagy (Mihaylova and Shaw^ 2011^; Kim et al. 2011). It has been reported that the PRKAA/AMPK-ULK1 axis is linked to ferritinophagy induced by metal particles in vascular endothelial cells (Qin et al. 2021). Our previous studies have found that senescent fibroblast cells exhibit impaired iron metabolism and lipid peroxidation, which could lead to ferroptosis-insensitive (Wei et al. 2023). We also found that the ferroptosis-insensitive in senescent fibroblast cells is closely related to the decrease in NCOA4 expression. Therefore, we hypothesized that AMPK activation can induce ferroptosis in senescent cells, and this process is related to NCOA4-mediated ferritinophagy.

In the present study, we tested the positive effects of AMPK activation on eliminating senescent cells and accelerating wound healing in diabetic mice. In our in vitro study, we observed that AMPK activation triggered ferroptosis in senescent fibroblast cells (SFBs). In addition, we revealed that AMPK activation triggered autophagy, which was directly linked to AMPK activation-induced ferroptosis. Furthermore, we demonstrated that AMPK-induced ferroptosis is autophagy-dependent, and this process is associated with NCOA4-mediated ferritinophagy. Hence, our study revealed the relevant mechanism of AMPK activation in reducing senescent cells, offering new therapeutic insights into diabetic wound healing.

## Material and methods

### Reagents

The chemical reagents A769662, Rapamycin and Z-VAD(OMe)-FMK were purchased from Cell Signaling Technology (USA).

Bafilomycin A1 (Baf A1), chloroquine (CQ) and deferoxamine mesylate salt (DFO) were purchased from Sigma-Aldrich (USA).

Anti-ARA70 and anti-ACSL4 antibodies were purchased from Santa Cruz Biotechnology (USA).

Anti-p62/SQSTM1, anti-AMPK and anti-pAMPK antibodies and horseradish peroxidase (HRP)-conjugated secondary antibody were purchased from Proteintech (USA).

4′,6-diamidino-2-phenylindole(DAPI), RIPA lysate buffer, SA-βgal staining kit,

BCA assay kit, ROS assay kit, JC-1 assay kit and GSH assay kit were purchased from Beyotime (Shanghai, China).

Anti-LC3B, anti-Ferritin Heavy Chain, Anti-Glutathione Peroxidase 4, anti-beta Actin and anti-GAPDH antibodies were purchased from Abcam (USA).

Real-time quantitative PCR (RT-qPCR) amplification primers for β-actin, AMPK, p-AMPK, GPX4, ACSL4 and SLC7A11 were synthesized by TsingkeBiotechnology (Beijing).

Cell Counting Kit-8 (CCK-8), malondialdehyde (MDA) assay kit and FerroOrange assay kit were purchased from Dojindo (Japan).

Dulbecco’s modified Eagle’s medium (DMEM) was purchased from Gibco (USA). Fetal bovine serum was purchased from Biological Industries (ISL).

### Isolation and culturing of primary mouse skin fibroblast cells

Primary mouse skin fibroblast cells (FBs) were isolated according to the method described in our previous study (Wei et al. 2023). The skin tissue of suckling mice was taken and minced. Digestive enzymes were added for 8 h, and the FBs suspension was obtained after centrifugation.

FBs were cultured in DMEM supplemented with 10% fetal bovine serum and 0.5% streptomycin. Then, primary FBs were cultivated in a high glucose (33.3 mM) complete medium for 14 days to develop senescent fibroblast cells (SFBs). Cells were incubated in a humidified atmosphere containing 5% carbon dioxide at 37 °C.

### Cell viability

Cell viability was measured using a Cell Counting Kit-8 following the manufacturer’s instructions. Cells were seeded in 96-well plate at 1 × 10^4^ cells per well. After overnight growth, cells were treated with A769662 for 24h or 48h, and 10 μL of CCK-8 reagent was added to each well for an additional 1h incubation. Absorbance at 450 nm of each well was measured using a microplate reader.

### Measurement of ROS and MDA

Total intracellular ROS levels were measured using the redox-sensitive fluorescent dye 20,70 -dichloroflfluorescin diacetate assay kit according to the manufacturer’s instructions.

The cell lysates or tissue lysates were measured using an MDA detection kit according to the manufacturer’s instructions.

### Determination of total intracellular iron

Total metal concentration was measured using the FerroOrange assay kit. In brief, the cells were seeded in fluorescent dishes and cultured overnight. Then, the cells were washed with serum-free medium for 3 times before being harvested with 1 µmol/L FerroOrange working solution for 30 min at 37 °C. After incubation, the cells were observed under a fluorescence microscope.

### Senescence-associated β-galactosidase assay in cells and tissues

SA-βgal staining of cultured cells was measured by assay kit. Cells were seeded in a 6-well plate and then incubated overnight. Cells were incubated with 1 ml of β-galactosidase staining fixation solution, and fixed for 10 min at room temperature. Washing cells with PBS for 3 times, and then cells were incubated with 1 ml working solution for 2 h at 37 °C. The cells were observed under a light microscope.

SA-βgal staining of the tissue was performed as protocol described previously. Skin tissue harvested from diabetic mice was promptly frozen in liquid nitrogen and then stored at − 80 °C. Tissue specimens were set in cryostat section disks with optimal cutting temperature compound (OCT). Then, the cryostat was cut into 7 µm sections at − 25 °C and immediately mounted on resin slides. These slides were fixed with β-galactosidase staining fixation for 10 min at room temperature. The tissue sections were washed with PBS for 3 times and incubated with β-galactosidase staining fixation working solution overnight. Then dehydrating the sections with ethanol and stain them with eosin (2%) for 3 min. The sections were observed under a standard light microscope.

### Western blot analysis

Cells and tissues were lysed using RIPA lysis buffer containing a protein phosphatase inhibitor. The lysates were then incubated on ice for 15 min before being centrifuged at 13,000 rpm for 20 min at 4 °C. The supernatant was collected in another tube after sonication in an Ultrasonic Cell Disruptor (amplitude setting 2, two cycles of 15 s). The protein concentrations of cell lysates were determined by the BCA Assay Kit according to the manufacturer’s instructions. Proteins were separated via SDS-PAGE and transferred onto polyvinylidene fluoride (PVDF) membrane. The membrane was blocked with 5% (w/v) skimmed milk at room temperature for 2h. The membrane was incubated with primary antibodies at 4°C overnight and then with secondary antibodies. The following antibody dilutions were used: Anti-p62/SQSTM1 (1:2000), anti-AMPK (1:2000), anti-pAMPK (1:2000), anti-LC3B (1:500), anti-Ferritin Heavy Chain (1:1000), anti-Glutathione Peroxidase 4 (1:1000), anti-beta Actin (1:5000), anti-GAPDH (1:10,000), anti-ARA70 (1:100) and anti-ACSL4 (1:100). The antibody-reactive bands were identified by enhanced chemiluminescence kits.

### Immunofluorescent staining of cells

SFBs were seeded in a 12-well plate, treated with the indicated reagents, fixed with 4% paraformaldehyde for 30 min, incubated with 0.5% Triton X-100 for 10 min, and then further blocked with 1% BSA for 1 h. Cells were probed with primary antibodies overnight at 4 °C, followed by incubation with Alexa 488 and 592-labeled secondary antibodies for 1 h. Fluorescence images were taken under the Nikon fluorescence microscope.

### Measurement of mitochondrial membrane potential (MMP)

The MMP of cells was measured by a JC-1 assay kit. In brief, cells were seeded in 6 well plate and then incubated at 37 °C overnight. The cells were incubated with 1 ml of JC-1 staining solution for 20 min at 37 °C. During the incubation, prepare an appropriate amount of JC-1 staining buffer and placed on ice. After the incubation, wash cells with a buffer solution twice. The cells were observed under the Nikon fluorescence microscope.

### Cell transfection

NCOA4 small interfering (siRNA) and control-siRNA oligonucleotides for mouse species were synthesized by Obio Technology (Shanghai, China) (Table 1). SFBs were seeded in 6-well plates overnight and then transfected with control siRNA or NCOA4 siRNA using Lipo3000. After 6 h, the medium was changed, and the cells were treated with A769662 for 24 h at 48 h post-transfection.

**Table 1:** The sequence of primers

Primer	Sequences
Mouse-NCOA4	Sense: 5′-CCUCAAGUAUUGGGCCUUUTT-3′
	Antisense: 5′-AAAGGCCCAAUACUUGAGGTT-3′
Negative control	Sense: 5′-UUCUCCGAACGUGUCACGUTT-3′
	Antisense: 5′-ACGUGACACGUUCGGAGAATT-3′

### RT-qPCR

Total RNA was extracted using Trizol reagent (Leagene, Beijing, China). cDNA was synthesized using RT Master Mix (AGbio, Hunan, China). Realtime PCR was performed with SYBR Green fluorescence PCR kit (AGbio, Hunan, China) on QuantStudio™ Real-Time PCR System (ABI, USA). Relative mRNA levels were standardized against beta actin mRNA levels and determined using the 2^−∆∆CT^ method. The specific primers were synthesized by Tsingke (Beijing, China) and the detailed sequences were as follows:

Mouse *NCOA4* forward, 5′-GTGTGCTTGGAGAGACTGGG-3′;

Mouse *NCOA4* reverse, 5′-GCCACTGGATGCTGACTTCT-3′;

Mouse *p16* forward, 5′-GAACTCGAGGAGAGCCATCT-3′;

Mouse *p16* reverse, 5′-TGCCCATCATCATCACCTGAATC-3′;

Mouse *p21* forward, 5′-AGCAGAATAAAAGGTGCCACA-3′;

Mouse *p21* reverse, 5′-CATGAGCGCATCGCAATCAC-3′;

Mouse *p53* forward, 5′-ATCCTGGCTGTAGGTAGCGA-3′;

Mouse *p53* reverse, 5′-CCATGGCAGTCATCCAGTCT-3′;

### Mice diabetes model

The animal study was reviewed and approved by the Animal Experimentation Ethics Committee of Nanfang Hospital, Southern Medical School, China. The experimental protocols followed the guidelines outlined in the National Institutes of Health’s Guide for the Care and Use of Laboratory Animals. Male C57BL/6 mice were purchased from the Experimental Animal Center of Southern Medical University. All mice were housed in the Experimental Animal Center of Southern Medical University, maintained at 23 ± 1 °C (50 ± 5% relative humidity) with 12 h light/dark cycles with free access to water and a regular chow diet. For the STZ model, 6–8 weeks mice were randomly divided into two groups. One group of mice was intraperitoneally injected with streptozocin (50 mg/kg body weight, 0.1 M citrate buffer, pH 4.5) to induce diabetes for 5 consecutive days, and the other group of mice was intraperitoneally injected with the same volume of citrate buffer. Blood glucose levels were checked every 2 days using an Accu-Check Active glucometer (Roche, Lyon, France) after the STZ injection. Mice were considered to have type 1 diabetes when blood glucose was ≥ 300 mg/dL for 3 consecutive days.

### Skin wound model

When all mice were considered to have diabetes, we proceeded to build the full-thickness wound model. We applied a splint model in all skin wound experiments. Mice were anesthetized by inhaling 3% isoflurane. Prior to excision for wounds, the dorsal hair was shaved with an electric clipper, followed by a depilatory cream. The skin was rinsed with alcohol, and two full-thickness wounds were created on the dorsum on each side of the midline, using a 10-mm biopsy punch. As previous study described, the silicone ring is tightly sutured to the skin around the wound site (Wang et al. 2013). Then, a sheet of Tegaderm™ (3 M Medical, Diegem, Belgium) was cut in a proper size and stuck on the back of the mice. Digital photographs were taken on the day of surgery and on days 0, 2, 4, 7, and 14, after the skin wound. Normal saline (NS) and A769662 (20 μM) were applied topically using pipettes onto the wound beds at 100 μL per time. The volume of agents applied was set according to the wound area. Agents were administered every other day. In day 14, we sacrificed the mouse and collected the skin tissue from the wound site and surrounding area (approximately 1 cm diameter) for subsequent analysis.

### In vivo wound healing assessment

On days 0, 2, 4, 7, and 14, after skin wounding, the patches were removed and images were acquired using a digital camera. Wound areas were measured using ImageJ software, and the percentage wound closure (C%) was calculated as in.

1 $${\text{C}}\% =\left[ {\left( {{\text{C}}_0 - {\text{C}}}_t \right)/{\text{C}}}_0 \right] \times 100\%$$

where C_0_ and C_t_ are the wound areas at day 0 and each timepoint, respectively.

### Histological analysis

On day 14, after skin wounding, all mice were euthanized by pentobarbital sodium overdose, and the wound tissues were excised and fifixed in 4% paraformaldehyde. The samples were dehydrated, embedded in paraffifin, sliced into 7-μm-thick sections, deparaffifinized with xylene, and rehydrated with ethanol. Tissues sections were stained with hematoxylin and eosin (HE) and Masson staining following the manufacturer’s protocol.

### Statistical analysis

All experiments were repeated at least three times, and the data are presented as means ± standard deviation (SD). Statistical analysis was performed using a Prism 9.0 software (GraphPad, La Jolla, CA). Two-group and multigroup comparisons were performed with Student’s t test and one-way ANOVA, respectively. A P value < 0.05 was considered statistically signifificant.

## Results

### AMPK was a crucial gene in high glucose induced senescent fibroblasts

**Fig. 1:**
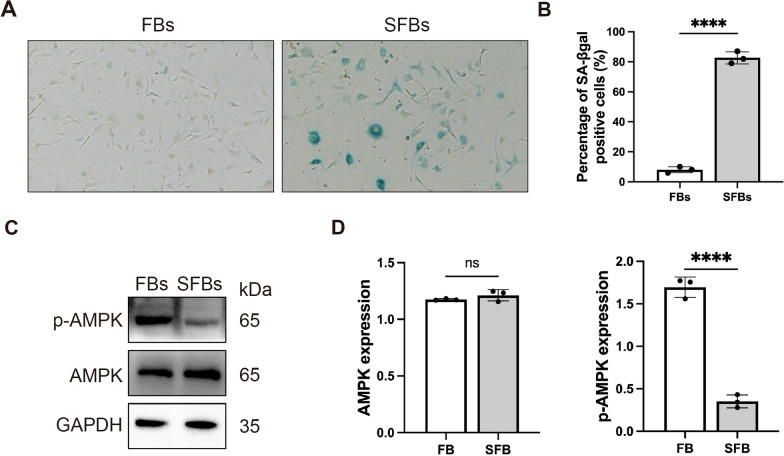
AMPK is a crucial gene in high glucose induced senescent fibroblasts. **A** and **B** SA-βgal staining analysis in fibroblast cells and HG-induced senescent fibroblast cells. The senescent fibroblast cells are staining positive for SA-βgal activity (blue staining). Statistical analysis of SA-βgal positive cells was shown. **C** and **D** Western blotting analysis of p-AMPK and AMPK expression in FBs and SFBs. Statistical analysis of densitometry was shown. GAPDH was detected as a loading control. Statistical analysis was performed by Student’s t-test: significant (*p < 0.05, **p < 0.01, ***p < 0.001 and ****p < 0.0001). The mean ± SD are shown; n = 3 independent experiments

We initially studied a model of cellular senescence in mouse skin fibroblasts (SFBs) induced by high glucose (HG). After HG treatment, we detected the level of SA-βgal (the marker of cell senescence) positive cells (Fig. 1A). The SA-βgal staining results showed that HG treated cells had senescence development.

AMPK, as a key molecule in cell metabolism, plays a vital role in cellular senescence (Ge et al. 2022). Some studies have found that in some aging-related diseases, AMPK pathway is suppressed, which can influence cell function (Kimura et al. 2020; Ju et al. 2011; Zhang et al. 2017). Herein, we explored that whether AMPK activation is downregulated in HG-induced SFBs. We detected the protein expression of p-AMPK in FBs and SFBs. The results of western blot assay suggested that the SFBs group expressed decreased p-AMPK level and p-AMPK/AMPK ratio following HG treatment (Fig. 1C and D).

### AMPK activation can reduce cellular senescence of fibroblast

**Fig. 2:**
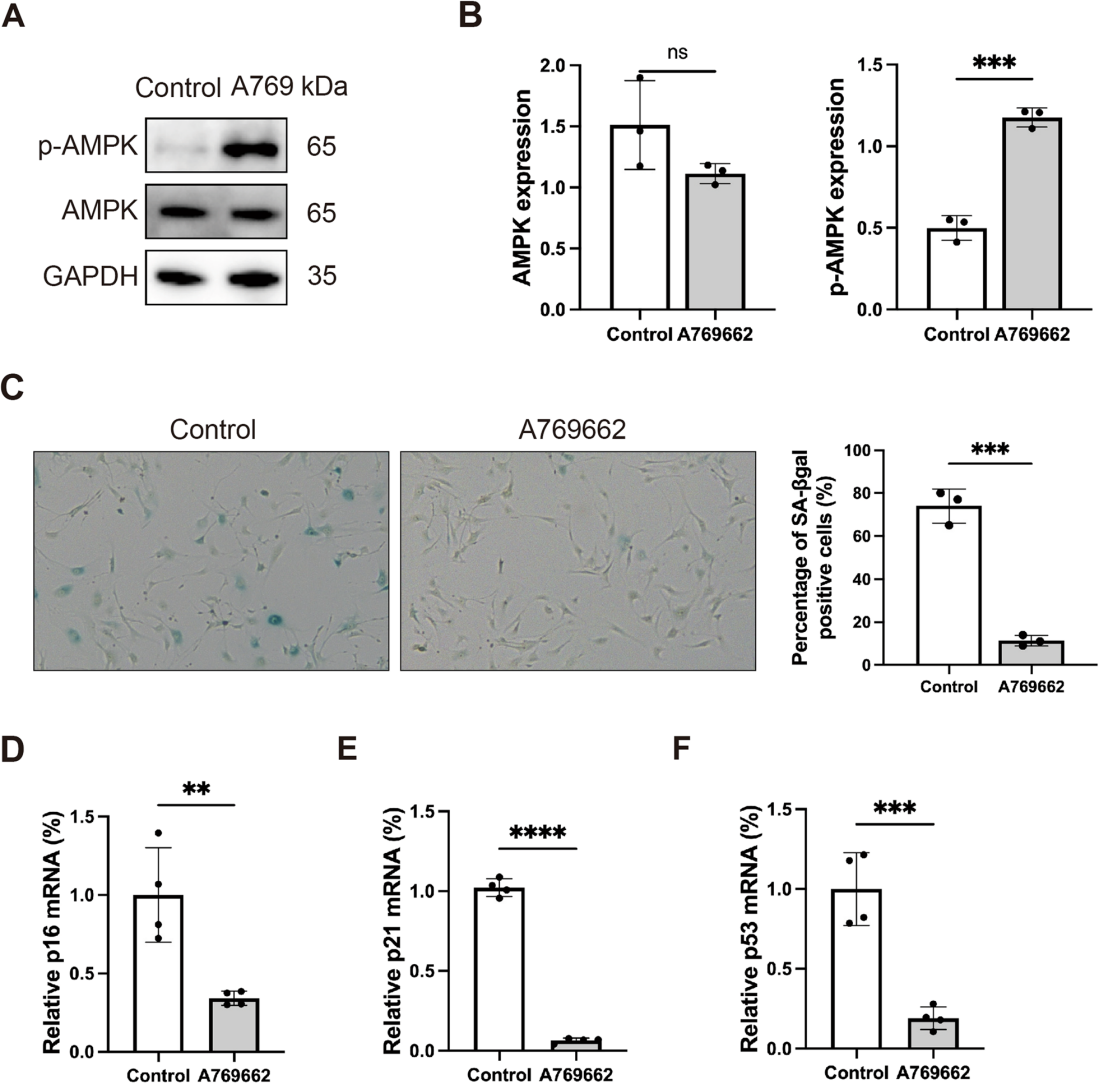
AMPK activation can reduce cellular senescence of fibroblast. **A** and **B** Western blotting analysis of p-AMPK and AMPK expression in SFBs and SFBs treated with A769662 for 24 h. GAPDH was detected as a loading control. Statistical analysis of densitometry was shown (n = 3 independent experiments). **C** Percentage of senescent cells following A769662 (30 µM) treatment. The senescence cells are staining positive for SA-βgal activity (blue staining). Statistical analysis of SA-βgal positive cells was shown (n = 3 independent experiments). **D** p16 mRNA expression as determined by RT-PCR (n = 4). **E** p21 mRNA expression as determined by RT-PCR (n = 4). **F** p53 mRNA expression as determined by RT-PCR (n = 4). Statistical analysis was performed by Student’s t-test: significant (*p < 0.05, **p < 0.01, ***p < 0.001 and ****p < 0.0001). The values are expressed as the mean ± S.D

Having established that AMPK activation is suppressed in senescent cells, we treated SFBs with A769662, a molecule directly targeting the AMPK receptor, to activate AMPK in SFBs (Cool et al. 2006). We detected cell viability after A769662 treating with 0, 20, 30, 40, 50, 60 or 70 µM A769662. The CCK8 results showed that A769662 could induce the death of SFBs in a dose-dependent manner (Figure S1). In particular, SFBs displayed a significant decrease in cell viability after treatment with 30µM A769662. Therefore, we chose 30µM for further experiments. After A769662 treatment, the percentage of p-AMPK is significantly increased (Fig. 2A and B). Then, we detected the cellular senescence of A769662 treated cells by SA-βgal staining (Fig. 2C). The HG-induced SFBs stained positive for more than 75%, while in A769662 treated group less than 20% SFBs stained positive. Furthermore, we detected the expression of p16INK4a and p21-Cip1, which are highly recognized markers of cellular senescence (Sharpless and Sherr 2015). p21 can universally bind receptors and hinder the production of various cyclin-dependent kinase (CDK) complexes, impede the production of DNA, and consequently cause cells to stagnate in the G1 phase, and eventually induce cellular senescence (Kumari and Jat 2021). The p53 gene is a crucial gene associated with cell growth, aging and DNA repair. Studies have pointed out that reducing the expression of p53 can alleviate skin aging (Strozyk and Kulms 2013). RT-PCR analyses revealed that in A769662 treated group the mRNA levels of p16, p21 and p53 are decreased (Fig. 2D–F). Taken together, these results suggest that AMPK activation can eliminate senescence cells in high sugar condition.

### AMPK activation can promote wound healing

**Fig. 3:**
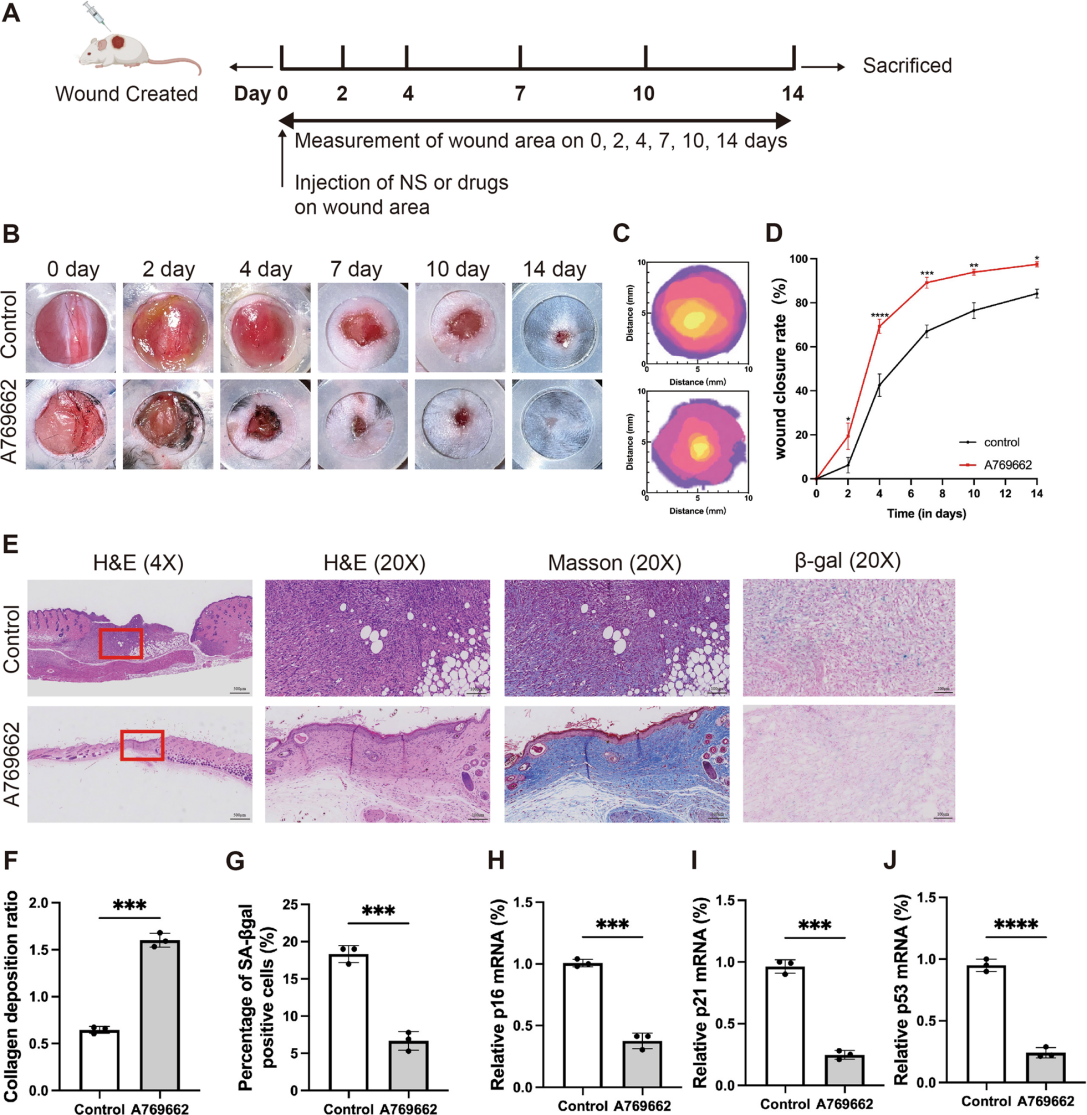
AMPK activation can promote wound healing and reduce tissue senescence. **A** The experimental design of in vivo study. The detailed information was described in the Materials and Methods section. **B** Photographs of wound tissues treated with different treatment groups on day 0, 2, 7, 10and 14. **C** Wound healing boundaries in different treatment groups in vivo. **D** Wound closing rate in different treatment groups at different time points (n = 3 per group). **E** H&E staining, Masson staining and SA-βgal staining of the wound indicated the healing situation on day 14. For Masson staining, the blue areas showed newly synthesized collagen fibers. The senescence cells are staining positive for SA-βgal activity (blue staining). **F** Quantitative analysis of collagen deposition by Masson staining (n = 3). **G** Quantitative analysis of SA-βgal positive cells (n = 3). **H** p16 mRNA expression as determined by RT-PCR (n = 4). **I** p21 mRNA expression as determined by RT-PCR (n = 4). **J** p53 mRNA expression as determined by RT-PCR (n = 4). Statistical analysis was performed by Student’s t-test (**F**–**J**) or two-way ANOVA (**D**): significant (*p < 0.05, **p < 0.01, ***p < 0.001 and ****p < 0.0001). The values are expressed as the mean ± S.D

To investigate whether activating AMPK can clear senescent cells in vivo and promote wound healing, we induced type 1 diabetes in mice via intraperitoneal (i.p.) injecting streptozotocin (STZ). When the blood glucose of mice was maintained at a high level (≥ 300 mg/dL) for 3 consecutive days, we created full-thickness skin defects in diabetic mice to assess the wound healing potential of AMPK activation (Fig. 3A). An untreated group (only treated with normal saline) was taken as the control group. We topically injected A769662 on the wound site to activate AMPK. The representative images displayed the healing progress on days 0, 2, 4, 7, 10 and 14 (Fig. 3B). The images of the wound area at different times are quantitatively analyzed (Fig. 3C). On day 4, the A769662-treated group exhibited a higher wound healing rate than the control group. This indicates the positive effect of AMPK activation on wound healing. On day 14, the wound in A769662-treated group was almost healed, and the wound healing rate is higher than the control group (Fig. 3D). These results showed that the A769662 group exhibited a significant increase in wound closure. H&E staining was performed to observe the granulation and cellular migration, and Masson staining was performed to detect collagen deposition (Fig. 3E). A769662-treated group exhibited a complete epithelial structure, indicating an acceleration of epithelialization process. Additionally, the collagen deposition ratio is much higher in A769662-treated group (Fig. 3F). Further, we evaluated the effect of reducing cellular senescence through AMPK activation. The results of SA-βgal staining showed that the A769662-treated group exhibited a lower amount of SA-βgal positive cells than the control group (Fig. 3E and G). The results of mRNA expression levels of p16, p21 and p53 showed that A769662-treated group exhibited a lower expression of p16, p21 and p53 mRNA (Fig. 3H–J). All these indicators confirmed that AMPK activation has the potential of reducing cellular senescence in DW.

### AMPK activation induced ferroptosis in SFBs

Recent studies have found that AMPK activation has a close relationship with ferroptosis (Han et al. 2020; Song et al. 2018). Ferroptosis is a new type of programmed cell death mode, which is triggered by iron overload and lipid peroxidation (Dixon et al. 2012). Our previous studies have found that senescent fibroblasts are resistant to ferroptosis (Wei et al. 2023).

**Fig. 4:**
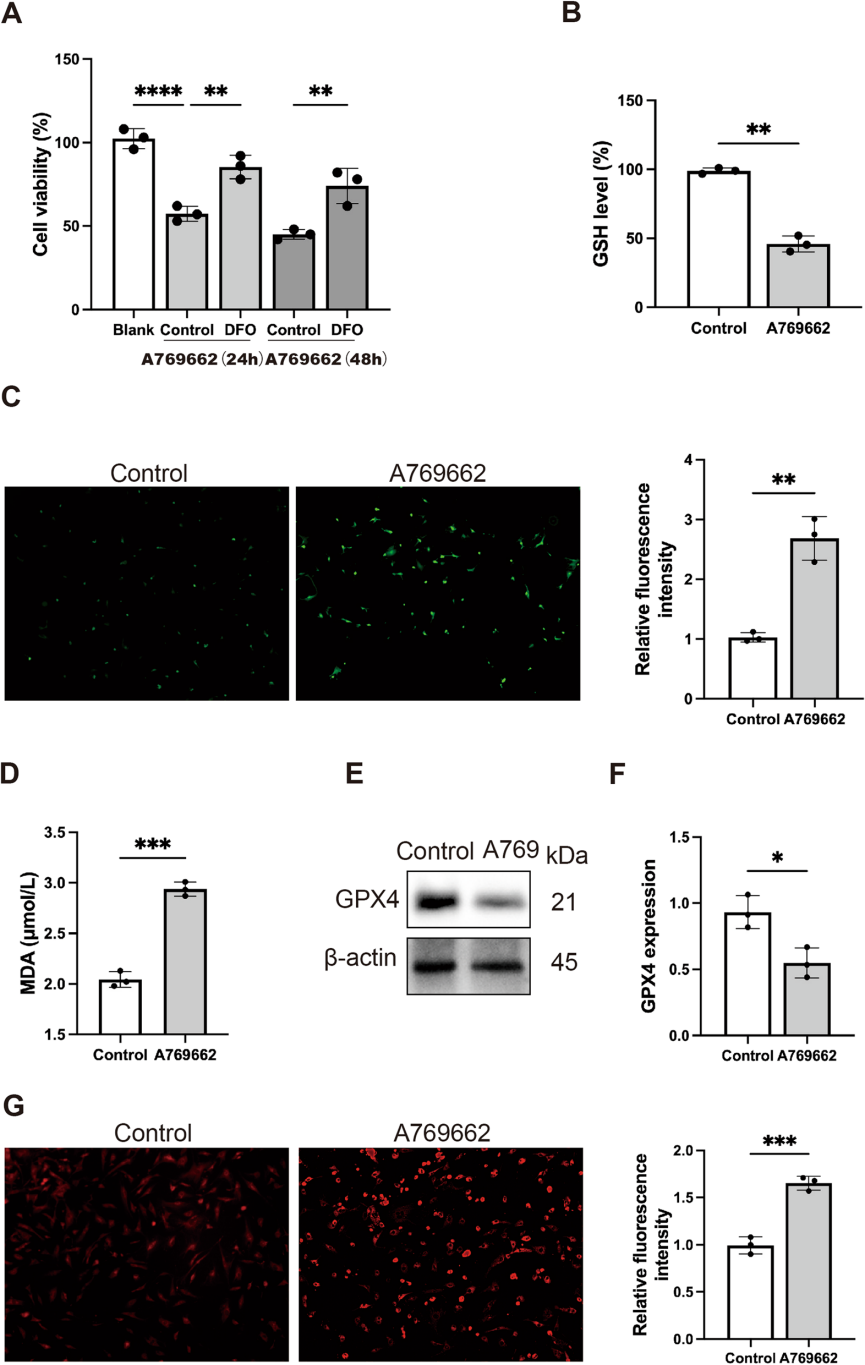
AMPK activation induced ferroptosis in SFBs. **A** CCK8 analysis of SFBs following treatment with A769662 (30 µM) and DFO (10 µM) for 24 h or 48 h (n = 3). **B** GSH level analysis in SFBs following A769662 (30 µM) treatment for 24 h were determined. Statistical analysis of GSH level was shown (n = 3). **C** ROS level ananlysis in SFBs following A769662 (30 µM) treatment for 24 h were determined. Statistical analysis of fluorescence densitometry was shown. n = 3 randomly selected fields from 3 independent experiments. **D** MDA content in SFBs following A769662 (30 µM) treatment for 24 h were determined (n = 3). **E** and **F** Western blotting analysis of GPX4 expression in SFBs and SFBs treated with A769662 for 24 h. β-actin was used as an internal control. Statistical analysis of densitometry was shown (n = 3). **G** Intracellular iron level in SFBs following A769662 (30 µM) treatment for 24 h were determined. Statistical analysis of fluorescence densitometry was shown. n = 3 randomly selected fields from 3 independent experiments. All data were analyzed by Student’s t-test and the values are expressed as the mean ± S.D. *p < 0.05, **p < 0.01, ***p < 0.001 and ****p < 0.0001

Our research has shown that AMPK activation can induce cell death. Then, we investigated whether inhibition of ferroptosis can prevent the cell death induced by AMPK activation. DFO is a chelator of iron, and it can suppress the process of ferroptosis (Dayani et al. 2004). The CCK8 assay revealed that DFO pretreatment significantly alleviated A769662-induced death of SFB cells (Fig. 4A), indicating that ferroptosis was involved in SFB cell death induced by A769662.

GSH is an important antioxidant that plays a key role in the redox balance within cells. The ferroptosis process can decrease the amount of GSH (Xu et al. 2022). The antioxidant system is inhibited due to the accumulation of intracellular iron, which can produce excess ROS through Fenton reaction (Jiang et al. 2021). Lipid peroxidation is also an important marker of ferroptosis (Li et al. 2019). So, we detected the GSH level, ROS level and MDA (malondialdehyde, a lipid peroxidation product) level after AMPK activation (Figure B, C, and D). The results showed that ROS and MDA contents were significantly increased in the A769662-treated group. We also measured the levels of GPX4, which is related to lipid peroxidation. The GPX4 level significantly decreased after A769662 treatment (Fig. 4E and F). Further, we investigated the intracellular iron levels of SFBs, and the results showed that after AMPK activation, the intracellular iron levels were higher than those in non-treated SFBs (Fig. 4G). Taken together, these results demonstrate that AMPK activation can induce ferroptosis in SFBs.

### AMPK activation triggered autophagy in SFBs

**Fig. 5:**
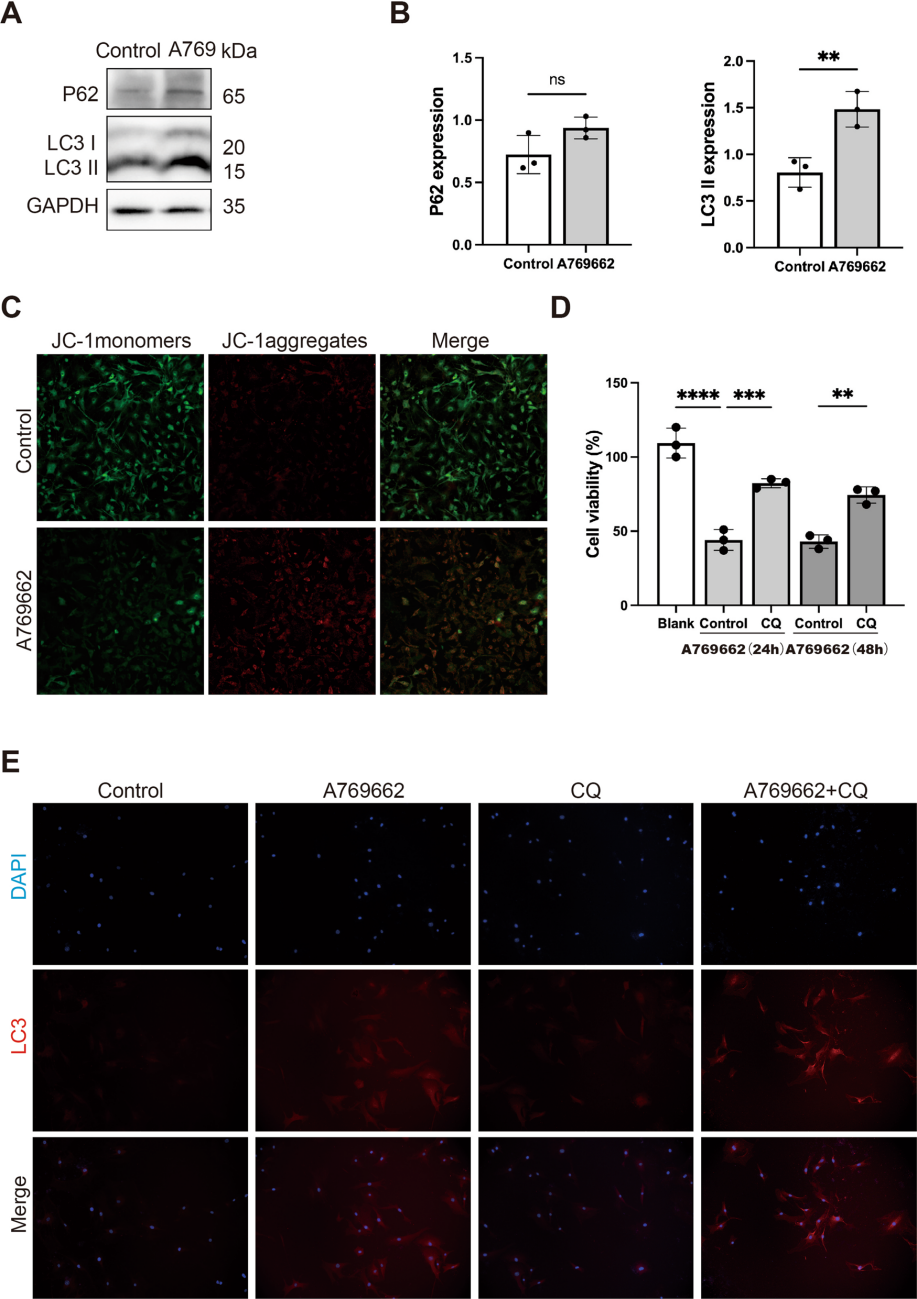
AMPK activation triggered autophagy in SFBs. **A** and **B** Western blotting analysis of LC3B-I/II and P62 expression levels in SFBs following A769662 (30 µM) treatment for 24 h. Statistical analysis of densitometry was shown (n = 3). **C** JC-1 assay showing mitochondrial membrane potential (MMP) level in SFBs and SFBs after A769662 treatment. **D** CCK8 analysis of SFBs following treatment with 30 µM or 40 µM A769662 and CQ (10 µM) for 24 h (n = 3). **E** Representative immunofluorescence images of LC3 (red) by fluorescence microscope. Nuclei were stained with DAPI (blue). All data were analyzed by Student’s t-test and the values are expressed as the mean ± S.D. *p < 0.05, **p < 0.01, ***p < 0.001 and ****p < 0.0001

AMPK plays an important role in the autophagy process (Kim et al. 2011; Garcia and Shaw 2017). Ferroptosis is considered as an autophagic cell death process (Gao et al. 2016). Thus, we investigated whether autophagy is activated in A769662-induced ferroptosis of SFBs. The western blot analysis results showed that A769662 treatment upregulated the protein level of LC3B-II (a marker of autophagy), while the expression of the autophagic substance p62 showed no significant change (Fig. 5A and B). We used the JC-1 probe to investigate the function of mitochondria. Compared with the control group, the red fluorescence ratio was higher in the A769662 treated-group, indicating that the mitochondrial membrane potential (MMP) of cells was conspicuously increased after AMPK activation (Fig. 5C). Then, we investigated whether inhibition of autophagy can prevent the SFBs death induced by AMPK activation. The CCK8 assay revealed that CQ pretreatment significantly alleviated A769662-induced death of SFBs (Fig. 5D), indicating that autophagy was involved in SFBs cell death induced by A769662.

As the accumulation of LC3B-II at a single time point does not reflect the change in autophagy, the LC3B-II protein level was detected with and without autophagy inhibitors. The immunofluorescence results showed that pretreatment with chloroquine (CQ) (Fig. 5E) further increased the LC3B-II protein level in A769662-treated SFBs, suggesting that AMPK activation triggers autophagy in SFBs.

### Ferritinophagy was involved in AMPK-triggered ferroptosis in SFBs

Ferritinophagy is the autophagic degradation of ferritin, which releases intracellular iron and promotes ferroptosis (Dowdle et al. 2014). And this process is mediated by NCOA4, a selective cargo receptor of ferritinophagy (Bellelli et al. 2016). We have previously demonstrated that activating AMPK can promote autophagy and induce ferroptosis. Herein, we investigated whether ferritinophagy is involved in AMPK activator induced ferroptosis.

**Fig. 6:**
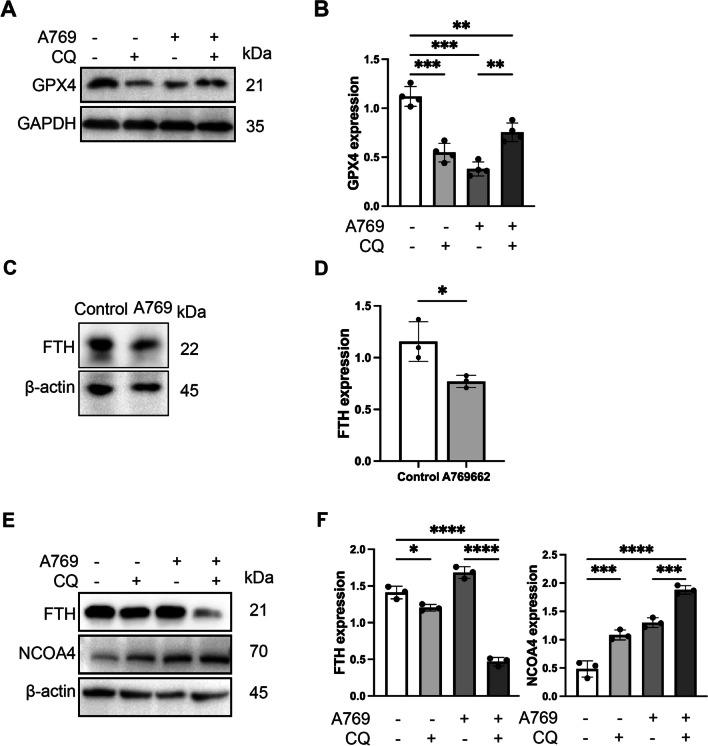
The ferritinophagy was involved in AMPK triggered ferroptosis in SFBs. **A** and **B** Western blotting analysis GPX4 expression levels in SFBs following A769662 (30 µM) treatment with or without CQ (10 µM) for 4 h (n = 3). Statistical analysis of densitometry was shown. **C** and **D** Western blotting analysis FTH expression levels in SFBs following A769662 (30 µM) treatment for 24 h (n = 3). Statistical analysis of densitometry was shown. (E and F) Western blotting analysis NCOA4 and FTH expression levels in SFBs following A769662 (30 µM) treatment with or without CQ (10 µM) for 4 h (n = 3). Statistical analysis of densitometry was shown. The data were analyzed by Student’s t-test (**D**) or one-way ANOVA (**B** and **F**). The values are expressed as the mean ± S.D. *p < 0.05, **p < 0.01, ***p < 0.001 and ****p < 0.0001

We measured the levels of GPX4 after AMPK activation following CQ treatment. Inhibition of autophagy with CQ significantly decreased the protein expression of GPX4 (Fig. 6A and B), which suggested that ferroptosis is associated with autophagy. The western blot analysis results revealed that A769662 treatment induced a decrease in FTH expression in SFBs (Fig. 6C and D). We speculated that the decrease in FTH may be due to the promotion of ferritin autophagy by AMPK activation. Then, we investigated the levels of NCOA4 and FTH, which are related to the process of ferritin degradation and iron metabolism. Inhibition of autophagy with CQ significantly decreased the level of FTH and increased the level of NCOA4 (Fig. 6E and F). Altogether, these results indicated that ferritinophagy is associated with ferroptosis induced by AMPK activation in SFBs.

### NCOA4 is essential for the ferroptosis induced by AMPK activator in SFBs

**Fig. 7:**
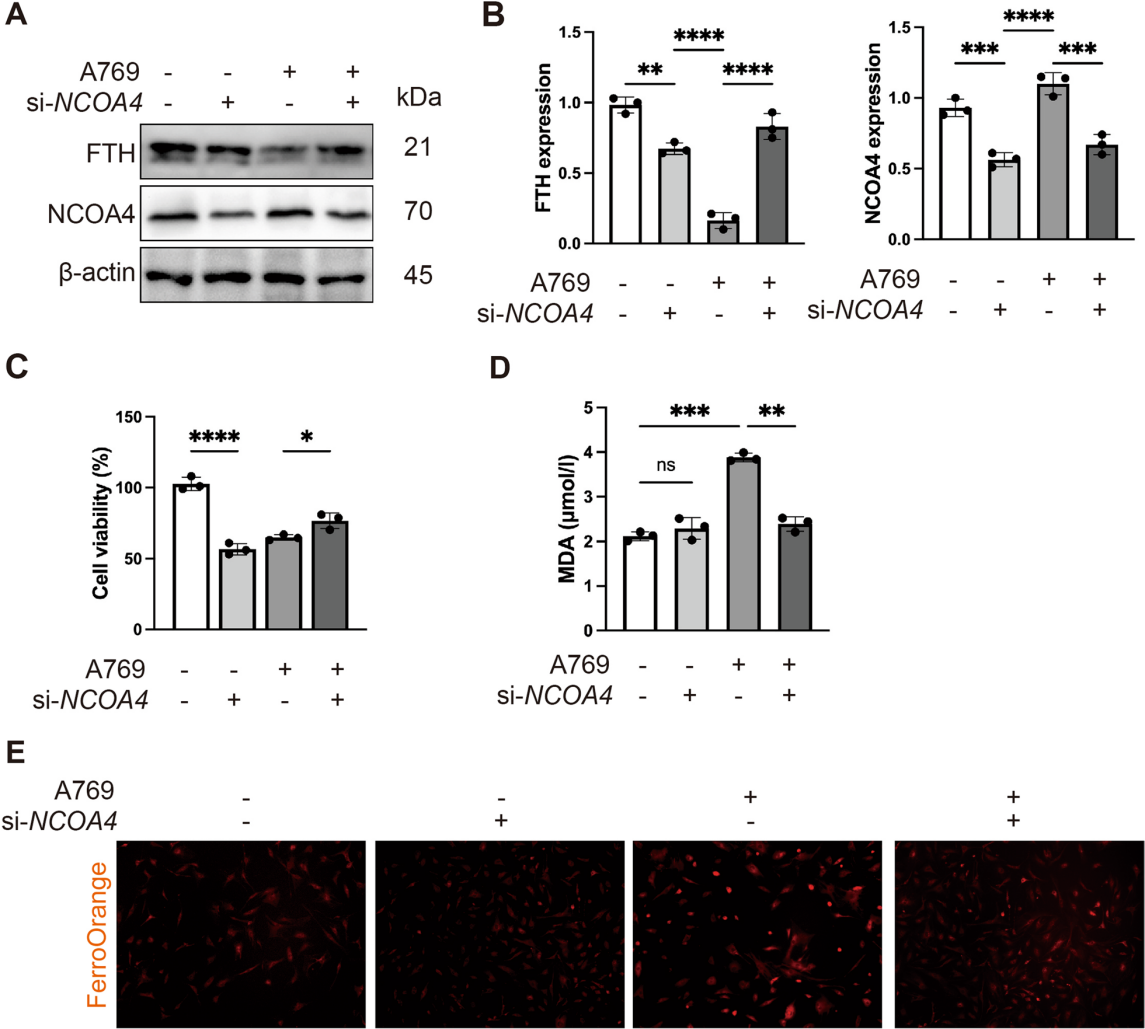
NCOA4 is essential for the ferroptosis induced by AMPK activator in SFBs. SFBs were transfected with specific NCOA4 siRNA (si-NCOA4) for 48 h before A769662 (30 µM) treatment. **A** and **B** Western blotting analysis NCOA4 and FTH expression levels in SFBs following A769662 (30 µM) treatment for 24 h (n = 3). Statistical analysis of densitometry was shown. **C** CCK8 analysis of SFBs following treatment with A769662 (30 µM) for 24 h (n = 3). **D** MDA contents in SFBs following A769662 (30 µM) treatment for 24 h (n = 3). **F** Intracellular iron level in SFBs following A769662 (30 µM) treatment for 24 h. n = 3 randomly selected fields from 3 independent experiments. All data were analyzed by one-way ANOVA. The values are expressed as the mean ± S.D. *p < 0.05, **p < 0.01, ***p < 0.001 and ****p < 0.0001

In order to identify the role of NCOA4 in ferroptosis induced by AMPK activation, we used siRNA to knock down NCOA4 in SFBs. After NCOA4 knock down measurement for 48 h, the cells were treated with A769662 for 24 h. The western blot results revealed that NCOA4 knockdown increased the expression level of FTH and decreased the expression level of NCOA4 (Fig. 7A and B), which suggested that ferritin degradation was inhibited by NCOA4 knockdown. Additionally, the CCK8 results showed that NCOA4 knockdown alleviated AMPK activation-induced cell death (Fig. 7C). Furthermore, the upregulation of MDA induced by the AMPK activator was also alleviated by NCOA4 knockdown (Fig. 7D). The FerroOrange assay revealed that NCOA4 knockdown reversed the upregulation of intracellular iron level induced by AMPK activator (Fig. 7E). These data indicate that NCOA4 mediated the degradation of FTH, releasing iron and promoting AMPK activation-induced ferroptotic death in SFBs.

## Discussion

Chronic non-healing wound is a common complication of diabetes. Understanding the molecular mechanisms involved in the development of chronic wounds is important for preventing diabetic ulcer. To address this issue, we developed an experimental model of diabetic wounds in mice. Several studies have shown that the excessive accumulation of senescent cells at the wound site delays the healing process and contributes to non-healing wounds (Blair et al. 2020; Wilkinson and Hardman 2020; Biran et al. 2017). Fibroblast senescence plays a main role in poor healing outcomes (Harding et al. 2005), and the number of existing senescent fibroblast cells is correlated with the healing time in chronic wound (Stanley and Osler 2001).

AMPK, as a main kinase regulating cellular homeostasis, plays a wide range of roles in cellular senescence and age-related diseases. Drugs that modulate the AMPK pathway have been widely used in diabetic patients (Madhavi et al. 2019). It was reported that Empagliflozin can improve the symptoms of diabetic mice by activating AMPK and alleviating mitochondrial fission (Zhou et al. 2018). In our study, we observed that the protein level of p-AMPK was significantly decreased in HG-induced SFBs (Fig. 1). These data suggested that AMPK plays a role in cellular senescence in diabetic wounds.

Recent studies have confirmed that cellular senescence is one of the main causes of diabetic non-healing wound (Wei et al. 2023). Hence, we want to investigate whether AMPK activation can remove senescent cells and accelerate diabetic wound healing. As expected, the SA-βgal activation increased after A769662 treatment, and the mRNA level of senescence marker p16 significantly decreased. Also, the markers related to cell cycle p21 and p53 were significantly decreased (Fig. 2). Our in vivo data confirm that AMPK activation treatment can remove senescent cells on the wound site and promote wound healing. As described in our study, the A769662 topical treatment exhibited the highest wound closing rate (Fig. 3). Interestingly, we observed that the A769662-treated group exhibited a more abundant extracellular matrix. In senescent cells, the extracellular matrix is degraded due to the over secretion of MMP9 (Ogrodnik 2021). Therefore, the change in extracellular matrix suggested that senescent cells were effectively removed. Also, the SA-βgal activation and senescence related markers p16, p21 and p53 significantly decreased in the A769662 group (Fig. 3).

Then, we explored the possible mechanism of AMPK regulating senescent cells death. Ferroptosis is a novel form of cell programmed death, which depends on intracellular iron and lipid peroxidation. Many studies are focusing on the role of ferroptosis in the metabolism of senescent cells (Zhao et al. 2021; Masaldan et al. 2018). Our previous studies suggested that senescent cells in diabetic wounds are ferroptosis-insensitive. In this study, we have confirmed that p-AMPK is decreased in SFBs and diabetic wound tissue. Therefore, we want to further explore whether AMPK is related to ferroptosis-insensitive in SFBs. Recent studies indicate that AMPK activity facilitates ferroptosis in Human trophoblastic cell and HUVECs (Han et al. 2020; Qin et al. 2021). We hypothesized that AMPK activation can induce ferroptosis in senescent cells. As expected, the protein level of ferroptosis marker GPX4 was significantly decreased in the A769662 treated group. The AMPK activation induces cell death, and this effect can be reversed by ferroptosis inhibitor DFO. AMPK activation also leads to lipid peroxidation and iron accumulation, which suggest that AMPK regulates the occurrence of ferroptosis (Fig. 4).

Recent studies have suggested that ferroptosis is an autophagy-dependent cell death (Hou et al. 2016; Angeli et al. 2014; Zhou et al. 2020). There are various autophagic pathways involved in the process of ferroptosis, such as NCOA4-mediated ferritinophagy, RAB7A-mediated lipophagy, mitophagy and HSP70-mediated CMA (chaperone-mediated autophagy). In western blot assay, we discovered a significant increase in the LC3B-II:I level in the A769662 treated group. Using autophagy inhibitor CQ to treat SFBs, we found that CQ can reverse the effect of A769662 on SFBs (Fig. 5). Our previous study has confirmed that NCOA4-mediated ferritinophagy is defected in SFBs. Therefore, we aim to figure out whether AMPK-induced autophagy is associated to ferritinophagy.

Ferritinophagy is an autophagic degradation process of ferritin, which is mediated by a selective cargo receptor NCOA4. In our study, we found that the protein levels of FTH and NCOA4 were in an inverse relationship during AMPK activation. Many studies have reported that NCOA4-mediated ferritinophagy is involved in various pathophysiological processes, such as erythropoiesis (Caulier and Sankaran 2022) and hepaticfibrosis (Zhang et al. 2018). The western blot assay suggested that FTH expression is significantly decreased in AMPK activated group. Along with the change in FTH levels, the expression of NCOA4 increased in AMPK activated group (Fig. 6). These results provided evidence that ferritinophagy is required for the ferroptosis-induced by AMPK activation.

Then, we detected the role of NCOA4 in AMPK activation induced ferroptosis process. We observed that NOCA4 knockdown can reverse the effects of AMPK activation (Fig. 7). Our study extends the role of NCOA4-mediated ferritinophagy in senescent cells in diabetic context.

## Conclusion

In summary, we reported that AMPK activation can eliminate senescent cells in the wound site and accelerate wound healing in diabetic mice. The eliminating effect of AMPK activation on SFBs was closely related to iron-dependent cell death. Moreover, we observed that the activation of ferroptosis was linked to NCOA4-mediated ferritinophagy. The current study extends the concept that AMPK has a positive effect on governing cell fate and provides an in-depth understanding of AMPK activation for biomedical applications. Collectively, AMPK activation may be a prospective method for removing senescent cells at diabetic wound sites and promote wound healing.

### Supplementary Information

Additional file 1.

## Data Availability

The authors confirm that all data in this study are fully available. All data generated or analyzed during this study are included in this published article and its supplemental information file.

## Abbreviations

AMPK: AMP-activated protein kinase
CQ: Choloroquine
DFO: Deferoxamine
FTH1: Ferritin heavy chain 1
GPX4: Glutathione peroxidase 4
GSH: Glutathione
LC3B: Light chain 3 beta
MTOR: Mechanistic target of rapamycin kinase
MDA: Malondialdehyde
NCOA4: Nuclear receptor coactivator 4
ROS: Reactive oxygen species
SQSTM1/p62: Sequestosome 1
TBS: Tris-buffered saline
