# Supplementary material for: AMPK activation eliminates senescent cells in diabetic wound by inducing NCOA4 mediated ferritinophagy
Source: Mol Med. 2024 May 17;30:63. doi: 10.1186/s10020-024-00825-8 (PMC11100200; doi:10.1186/s10020-024-00825-8)
Supplement: Supplementary file 1 — Additional file 1. [file 10020_2024_825_MOESM1_ESM.docx]

Supplement Information


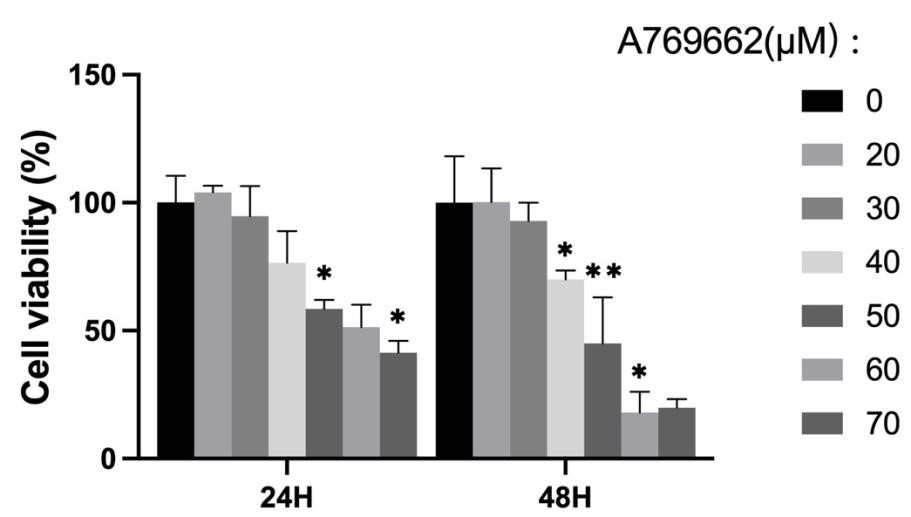


Supplement Figure S1. CCK8 analysis of SFBs treated with 0, 20, 30, 40, 50, 60 and 70µM A769662 for 24h and 48h.
